# Improving the neonatal mask ventilation skills of non‐airway experts using the “Five Breath Method”: A simulation study

**DOI:** 10.1002/pdi3.2520

**Published:** 2025-02-04

**Authors:** Anna Clebone

**Affiliations:** ^1^ School of Medicine University of Chicago Chicago Illinois USA

**Keywords:** mask ventilation, neonatal, neonatal resuscitation, pediatrics, resuscitation

## Abstract

Trainees in several medical disciplines are expected to achieve competence in neonatal resuscitation. Managing the neonatal airway requires faculties in mask ventilation. Many trainees, however, have limited clinical experience with this skill. An algorithmic method incorporating the steps an airway expert would use for neonatal mask ventilation was designed. The goal of teaching this system, the “Five Breath Method,” is to assist trainees with learning and incorporating the steps of neonatal mask ventilation into clinical practice. We hypothesized that for pediatric residents, participating in a teaching session on the Five Breath Method would significantly decrease the time to achieve ventilation (“air entry”) in a resuscitation scenario. A pilot study was completed on the Five Breath Method with 23 pediatric residents as subjects. Training in the Five Breath Method reduced the time required to achieve air entry using a neonatal simulator. Subjects required 57 [29–149 and 13–180] seconds (median [interquartile range and minimum–maximum]) before the teaching session and 16 [10–35 and 8–60] seconds after learning the Five Breath Method; *Z* = −4.11, and *P* < 0.001. For the 14 of the original subjects who were able to participate in the follow‐up study 2–5 months later, 24 [22–38 and 12–69] seconds (median [interquartile range and minimum–maximum]) were required to achieve air entry in the identical experimental scenario. This showed an improvement in time to air entry when compared to that before training; *Z* = −2.42, and *P* = 0.01552. This study showed that as little as 10 min of training could drastically improve skills. Learning the Five Breath Method also led to retention of skills over time.

## Graphical abstract

The most effective action in neonatal resuscitation is ventilation. A novel algorithm for mask ventilation, the “Five Breath Method”, incorporates steps that an airway expert would use, from least to most invasive. Training in the “Five Breath Method” leads to immediate improvement in mask ventilation and retention of this skill.

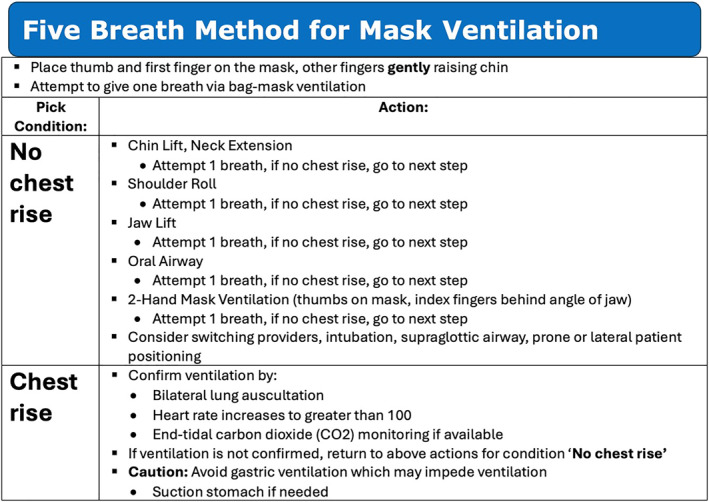

## 1 INTRODUCTION

According to the 2023 American Academy of Pediatrics (AAP)/American Heart Association (AHA) Guidelines for Neonatal Resuscitation, “Effective positive‐pressure ventilation is the priority in newborn infants who need support after birth”.^1^ Neonatal resuscitation seldom requires steps beyond ventilation. The underlying cause in up to half of the rare instances that require chest compressions or epinephrine (< 0.2% of all births) may be unsuccessful ventilation,^2^, ^3^ as “ventilation is the most effective action in neonatal resuscitation.”^4^

Only a small number of neonates (10%) “require some assistance to begin breathing at birth”^4^, so many trainees do not regularly have the opportunity to utilize mask ventilation in clinical situations. The American Academy of Pediatrics/American Heart Association advocates incorporating simulation‐based learning methodologies into resuscitation education.^4^

All current neonatal, pediatric, and adult resuscitation guidelines use an algorithmic approach to the steps of resuscitation. Successful mask ventilation involves a series of discrete maneuvers, which can be logically incorporated into a protocol. For these reasons, the “Five Breath Method” was designed as a stepwise, procedure‐based algorithm for the teaching and clinical practice of mask ventilation.^5^ A study was conducted to examine the application of the Five Breath Method to neonatal resuscitation using a high‐fidelity human simulator. For the primary aim, the hypothesis, that the time to achieve air entry via mask ventilation would decrease after participating in a brief teaching session on the Five Breath Method for pediatric residents in a resuscitation scenario, was tested. Secondarily, the hypothesis that residents would maintain this mask ventilation skill when retested over time was tested.

## 2 METHODS

### 2.1 The “Five Breath Method”

**FIGURE 1:**
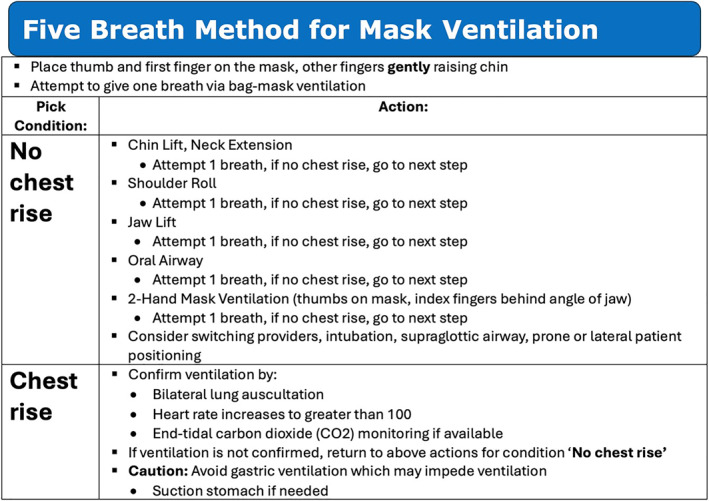
The “Five Breath Method” for mask ventilation.

The “Five Breath Method” for neonatal mask ventilation is a stepwise sequence of airway maneuvers intended to rapidly achieve air entry in a newborn patient who requires ventilatory support^5^ (Figure 1). The goal of the “Five Breath Method” is upper airway patency.^5^ Due to the fragile nature of the newborn, the steps required for difficult mask ventilation are placed in ascending order, from least to most invasive. This progression is designed to minimize the risk of causing airway trauma or triggering laryngospasm from an unnecessarily invasive step.^6^ After each step, the practitioner is asked to attempt to give one breath via bag‐mask ventilation, confirm ventilation, and return to airway maneuver steps if needed. Practitioners are directed to look for “chest rise” because this criterion is initially used to evaluate the patient for adequate ventilation in the AAP/AHA guidelines.^3^, ^7^ If chest rise is seen, the clinician is directed to confirm ventilation by auscultating the lungs bilaterally and confirming an increase in heart rate. If ventilation is not confirmed, the practitioner is directed to return to the “Five Breath Method.”

### 2.2 Study procedures

With Yale University Institutional Review Board approval (HIC#1009007412), physicians enrolled in a pediatric residency participated in this pilot study. The setting was a tertiary teaching hospital. Study procedures took place in an unused, empty, and clean patient or procedure room. Subjects were first tested with an experimental scenario. Next, subjects participated in a teaching session on the “Five Breath Method.” Finally, subjects were tested with the same experimental scenario a second time. All study procedures were performed by one researcher (Anna Clebone).

A neonatal mannequin (SimBaby, Laerdal, Wappingers Falls, New York, USA) on which airway maneuvers could be performed was used. An associated screen displayed the heart rate and oxygen saturation. A self‐inflating bag‐mask ventilation system was provided, similar to what was available for real‐world neonatal resuscitation in this setting. Subjects were oriented to the functions of the simulator and, as is standard for simulation scenarios, requested to “suspend disbelief” and take all actions as if the mannequin was a live patient.

The teaching session consisted of reviewing and practicing the “Five Breath Method.” During this teaching session, the method was reviewed twice with each subject. Any step that the subject was unfamiliar or uncomfortable with was demonstrated. The subject performed each step until they were able to demonstrate that step competently. This teaching session lasted 5–15 min, depending on the baseline skill level of each pediatric resident.

For the experimental scenario, subjects were told, “You have been called to a neonatal resuscitation; all other doctors were called away to another emergency. We have already dried the patient, rubbed his back, and cleared the airway, but the patient is apneic and blue, heart rate is 70 and oxygen saturation is 80%.” The investigator played the role of an inexperienced nurse and offered limited assistance. The SimBaby was programmed for complete airway obstruction (no air entry or endotracheal tube placement possible) until either a neck extension or jaw lift was performed. All scenarios were stopped after 3 min if air entry was not achieved.

At the end of these sessions, subjects were verbally debriefed with the question “what was similar/different about these neonatal resuscitation scenarios from patient encounters you previously participated in?” The researcher discussed these answers with the subject, and any questions about the steps of the “Five Breath Method” were answered. Pediatric residents completed a survey on previous experience and comfort level with mask ventilation.

### 2.3 Secondary study procedures

Subjects who participated in the primary study procedures were asked to return several months later to participate in secondary study procedures. This consisted of participation in the same experimental scenario, with the same procedures followed for mannequin settings, instructions, and location.

### 2.4 Sample size

The amount of time to achieve air entry was the primary outcome. After completion of the analysis, G*Power version 3.1.9.6^8^ was used to determine the needed sample size for the primary outcome. A 2 × 4 repeated measures analysis of variance showed that a sample size of at least 20 would be needed, with an effect size *f* = 0.6; power = 0.80; and significance level = 0.05 (without Bonferroni adjustment). The power of 80% is similar to that used for another study on simulated mask ventilation.^9^

### 2.5 Statistics

Data were analyzed via the Giga and Social Science Statistics calculators (web‐based, https://www.gigacalculator.com and https://www.socscistatistics.com), using methods similar to previous simulation studies evaluating time‐to‐task achievement. Time to air entry was the primary endpoint. Each subject served as their own control. The Shapiro–Wilk test was used to test for normality of distribution. A two‐tailed Wilcoxon signed‐rank test was used to compare time to air entry from pre‐training to post‐training. If air entry was not achieved within 3 min for each scenario, a time of 180 s was used for analysis purposes as “time to air entry.”

## 3 RESULTS

Twenty‐three pediatric residents completed all primary study procedures. Fourteen were in their first year of residency, 11 were in their second year of residency, and 1 was in his third year of residency. All residents had previous experience with neonatal simulation. Twenty‐one residents had previously performed fewer than five neonatal mask ventilation procedures in clinical situations, with only one resident previously performing more than 10 neonatal mask ventilation procedures. Three residents did not complete both scenarios due to technical malfunctions with the mannequin; data for these subjects were not included in the analysis.

Data for seconds to achieve air entry were not normally distributed (*P* < 0.005 and *W* = 0.7181). Our primary analysis showed that the time required to achieve air entry was 57 [29–149 and 13–180] seconds, median [interquartile range and minimum–maximum] before the teaching session and 16 [10–35, 8–60] seconds after learning the “Five Breath Method;” *Z* = −4.11, and *P* < 0.001.

Our secondary outcome was the retention of skills. For the 14 pediatric residents who completed the initial study procedures and were able to participate in the follow‐up study 2–5 months later, 24 [22–38 and 12–69] seconds, median [interquartile range and minimum–maximum] were required to achieve air entry in the experimental scenario. This showed an improvement in time to air entry when compared to that before training; *Z* = −2.42, and *P* = 0.01552.

## 4 DISCUSSION

Pediatric residents who received training in the novel “Five Breath Method” reduced the time required to achieve air entry using a neonatal simulator in a basic scenario. Before training, three residents were not able to successfully ventilate using a mask, whereas, after training, all residents were able to achieve air entry. This improvement persisted for those residents who participated in the experimental scenario again 2–5 months later. The observed immediate improvement to mask ventilation skills with this method is supported by the secondary outcome of the retention of these skills. The most effective action in neonatal resuscitation is ventilation. In the 2023 AAP/AHA guidelines, emphasis is placed on achieving ventilation by providing positive pressure breaths via bag‐mask ventilation.^1^ This novel algorithm for mask ventilation, the “Five Breath Method,” incorporates the steps that an airway expert would use, from least to most invasive.

Neonatal resuscitation is a core competency for many practitioners including pediatric residents and pediatricians.^10^ In one survey, almost a quarter of procedure‐performing pediatricians reported that staying proficient in infant bag‐mask ventilation was challenging.^11^ This problem is accelerated by pediatric residencies “de‐emphasizing the teaching and learning” of procedures, including airway skills.^10^ Our data suggest that employing a structured method, such as the “Five Breath Method,” could be beneficial for learning mask ventilation and retaining this skill.

The “Five Breath Method” is based on the steps that an expert might take to perform neonatal mask ventilation successfully. This cognitive aid directs the learner to begin with the least invasive airway maneuver and progress quickly yet incrementally toward more invasive steps if necessary. Recalling and executing simultaneous steps may be overwhelming to a practitioner who does not perform mask ventilation regularly. In contrast, a clinician who performs bag‐mask ventilation regularly may implement more than one of these steps simultaneously based on their assessment of the clinical situation.

In this study, retention of skills occurred when residents were retested 2–5 months later. This finding is consistent with other studies in which there was no difference in retention of newborn bag‐mask ventilation skills with retraining intervals of 1 month versus 3 months.^12^ Another broader study of neonatal resuscitation skills concluded that retraining should occur at least every 6 months.^13^ Although further research is needed, this is a reasonable conclusion based on existing evidence. This retraining should include updates based on local needs, equipment, and new evidence. Use of algorithms such as the “Five Breath Method” may also increase retention.

Adequate mask ventilation may be a better option than intubation for initially achieving ventilation in many neonates. In one study, only 62% of neonatal intubation attempts were successful, with many attempts taking more than 30 s.^14^ Less than 20 s for an intubation attempt is recommended by the Neonatal Resuscitation Program. Oxyhemoglobin saturation dropped in almost half of intubation attempts in which an oxygen saturation was measured.^14^ Adequate mask ventilation before intubation allows for additional alveolar oxygenation and therefore, more time before oxygen saturation decreases. In some neonates who require resuscitation, adequate mask ventilation may spare the need for intubation.

Training is the critical element in acquiring mask ventilation skills. We believe that the use of an algorithmic method is particularly suited to mask ventilation because of the discrete steps involved. When using an algorithm, decision‐making must occur in an organized, sequential manner.^15^ This is advantageous because a technique can then be “taught more effectively, monitored accurately, and understood better.”^15^

Few studies examine teaching of procedural skills or algorithms using simulation. Previous studies have instead used the simulator as a purely evaluative tool. We believe that the use of simulation as a teaching modality is an underexplored area in the research literature.

Worldwide, 10 million newborns will require resuscitation each year to successfully transition from fetal to neonatal circulation.^16^ Difficulties in neonatal mask ventilation are common. These difficulties are greater in preterm infants. One study showed that when providing positive pressure ventilation by bag‐mask to infants < 32 weeks gestational age, over half of clinicians delivered ventilations with a mask leak > 75%, and in a quarter of cases, significant obstruction occurred (defined in that study as a decrease in expired tidal volume of 75%).^17^

Prompt initiation of bag‐mask ventilation decreases mortality in the hypopnic or bradycardic newborn. In one large prospective study, for every delay of 30 s in beginning bag‐mask ventilation, the chance of death or prolonged admission increased by 16%.^18^

The study reported in this manuscript was carried out with a self‐inflating bag‐mask ventilation system, as that device was available for real‐world use in the study setting. A self‐inflating bag‐mask ventilation system has inherent limitations. With a self‐inflating bag‐mask ventilation system, there is a lack of feedback to the clinician as to whether an adequate seal is achieved between the patient's face and the mask and an inability to provide 100% oxygen unless ventilation is controlled. Additionally, a self‐inflating bag does not easily allow for spontaneous ventilation. In contrast, a flow‐inflating Mapleson system allows for spontaneous or assisted ventilation, for example, when airway obstruction is partially relieved with a jaw thrust or oral airway. However, a flow‐inflating system can only be used in settings where fresh gas flow is available; therefore, a self‐inflating system can be a backup in settings where additional fresh gas flow would not be available in case of emergency (e.g., an elevator). The American Heart Association and American Academy of Pediatrics guidelines suggest beginning positive pressure ventilation for neonatal resuscitation “with air (21% oxygen) in term and late preterm babies, and up to 30% oxygen in preterm babies.”^19^ These guidelines also recommend using a preductal oxygen saturation monitor to guide therapy, as newborn oxygen saturation is expected to be 60%–65% 1 min after birth and only rise to 85%–95% at 10 min after birth.^19^

This research has limitations. Subjects were from a single‐center residency program, so these data might not apply to other institutions and training situations. Additionally, a single task was used for the primary outcome, so the result could represent a learning effect. Nevertheless, all subjects had previous simulation experience, which suggests that a learning effect is unlikely.

Skills learned in a simulated environment do not necessarily translate to real‐world clinical practice. However, mannequins are widely used and accepted for airway training in pediatrics.^20^ Simulation‐based training is emphasized in the 2020 Neonatal Resuscitation guidelines.

This study did not fully replicate real‐world conditions because it involved a single subject performing a discrete skill. In real‐life newborn resuscitation, several clinicians perform tasks simultaneously. Team training has been shown to improve compliance with guidelines and the performance of clinical skills that are time‐critical.^20^ The performance of mask ventilation, however, often requires only a single practitioner, and this study was intended to teach that discrete skill.

Another limitation was that our study mostly involved first‐ and second‐year residents. Therefore, these results may not reflect what might be seen if third‐year residents, attendants, or other practitioners who may be involved in neonatal resuscitation were tested. Nevertheless, first‐ and second‐year residents are frequently involved in neonatal resuscitation. This result shows that the “Five Breath Method” is useful even for practitioners who are less experienced in their education and practice levels.

## 5 CONCLUSIONS

This study showed that a short training session could improve proficiency in neonatal mask ventilation. Further studies will be needed to determine if the “Five Breath Method” leads to greater skill retention over a longer period of time. Future research should also examine whether the “Five Breath Method” leads to improved mask ventilation during team simulation and improved outcomes during real‐world conditions.

## AUTHOR CONTRIBUTIONS

Dr Anna Clebone designed the protocol and scenarios, collected all data, performed the analysis, and wrote and approved this manuscript.

## CONFLICT OF INTEREST STATEMENT

The authors declare no conflicts of interest.

## ETHICS STATEMENT

This study was performed at Yale University and approved by the Yale University Institutional Review Board (HIC#1009007412).

## DATA PUBLICATION STATEMENT

These data have not been submitted elsewhere and have not been previously published in manuscript form. This research has been presented in abstract form at the Association for University Anesthesiologists and American Academy of Pediatrics Annual Meetings.

## Supporting information

Supplementary Material

## Data Availability

Data are available as “Supplemental Electronic Material” for this manuscript.
